# Costs of traditional Chinese medicine treatment for inpatients with lung cancer in China: a national study

**DOI:** 10.1186/s12906-022-03819-3

**Published:** 2023-01-09

**Authors:** Hanlin Nie, Zhaoran Han, Stephen Nicholas, Elizabeth Maitland, Zhengwei Huang, Sisi Chen, Zegui Tuo, Yong Ma, Xuefeng Shi

**Affiliations:** 1 School of Management, Beijing University of Chinese Medicine, Beijing, China; 2 Australian National Institute of Management and Commerce, Sydney, NSW Australia; 3 Guangdong Institute for International Strategies, Guangdong University of Foreign Studies, Guangzhou, China; 4 School of Economics and School of Management, Tianjin Normal University, Tianjin, China; 5 Newcastle Business School, University of Newcastle, Callaghan, NSW Australia; 6 University of Liverpool Management School, University of Liverpool, Liverpool, UK; 7 The Jockey Club School of Public Health and Primary Care, Faculty of Medicine, The Chinese University of Hong Kong, Shatin, Hong Kong, SAR China; 8 China Health Insurance Research Association, Beijing, China; 9 National Institute of Traditional Chinese Medicine Strategy and Development, Beijing University of Chinese Medicine, Beijing, China

**Keywords:** TCM, Lung cancer, Medical expenses, Joint clinical guideline

## Abstract

**Background:**

Traditional Chinese Medicine (TCM) has long been a widely recognized medical approach and has been covered by China’s basic medical insurance schemes to treat lung cancer. But there was a lack of nationwide research to illustrate the impact of the use of TCM on lung cancer patients’ economic burden in mainland China. Therefore, we conduct a nationwide study to reveal whether the use of TCM could increase or decrease the medical expenditure of lung cancer inpatients in mainland China.

**Methods:**

This is a 7-year cross-sectional study from 2010 to 2016. The data is a random sample of 5% from lung cancer claims data records of Chinese Urban Employee Basic Medical Insurance (UEBMI) and Urban Resident Basic Medical Insurance (URBMI). Mann-Whitney test was used to compare inpatient cost data with positive skewness. Ordinary least squares regression analysis was performed to compare the total TCM users’ hospitalization cost with TCM nonusers’, to examine whether TCM use is the key factor inducing relatively high medical expenditure.

**Result:**

A total of 47,393 lung cancer inpatients were included in this study, with 38,697 (81.7%) of them at least using one kind of TCM approach. The per inpatient medical cost of TCM users was RMB18,798 (USD2,830), which was 65.2% significantly higher than that of TCM nonusers (*P* < 0.001). The medication cost, conventional medication cost, and nonpharmacy cost of TCM users were all higher than TCM nonusers, illustrating the higher medical cost of TCM users was not induced by TCM only. With confounding factors fixed, there was a positive correlation between TCM cost and conventional medication cost, nonpharmacy cost (Coef. = 0.283 and 0.211, all *P* < 0.001), indicting synchronous increase of TCM costs and conventional medication cost for TCM users.

**Conclusion:**

The use of TCM could not offset the utilization of conventional medicine, demonstrating TCM mainly played a complementary role but not an alternative role in the inpatient treatment of lung cancer. A joint Clinical Guideline that could balance the use of TCM and Conventional medicine should be developed for the purpose of reducing economic burden for lung cancer inpatients.

## Background

Lung cancer is the second leading cause of new cancer cases worldwide. In 2020, there were 2.21 million new lung cancer cases, and 1.8 million lung cancer deaths, accounting for 11.4% of the new cancer cases and 18% of the cancer deaths [1]. Most lung cancer patients have a survival rate of only 10–20% at five years after diagnosis, which is very low compared with other major cancers, such as gastric cancer and liver cancer [1]. In China, lung cancer accounted for the greatest share, 25.4%, of disability-adjusted life-years (DALYs) for all cancer DALYs in 2019 [2]. In 2020, lung cancer caused 720, thousand deaths in China, accounting for 40% of the global lung cancer deaths [3], and imposed a huge economic burden on individuals, families, and China’s national health system [4]. A recent study showed that the average medical expenditure for lung cancer patients in China was 87,499 RMB ($13,173) in 2015–2016, which was much higher than China’s per capita disposable income (RMB23,819 / USD3,586 in 2016) [5], imposing catastrophic health expenditures on Chinese families.

Surgery, radiotherapy, chemotherapy, and targeted drug therapy have long been the major approaches to dealing with lung cancer [6–8]. However, the treatment costs and the concomitant side effects and morbidities, leads many lung cancer patients to complementary and alternative medicine (CAM) approaches. A large-scale survey in 14 European countries showed that 23.6% of lung cancer patients had used, at least once, CAM [9]. A report made by the University of North Carolina at Chapel Hill Lineberger Comprehensive Cancer Center in the United States revealed that 76.8% of lung cancer patients had received CAM treatment after diagnosis [10]. As a representative type of CAM, traditional Chinese medicine (TCM) plays a significant role in health maintenance in China and parts of East Asia [11–13]. TCM is the collective name of medicine of all nationalities in China, including Chinese and minority medicine, and is a system of medicine that reflects the Chinese nation’s understanding of life, health, and disease, with a long historical tradition and unique theories and technical methods. TCM includes many types of treatment methods, such as formulas, proprietary Chinese medicine, acupuncture, moxibustion, and Tuina, which are widely used in clinical practice. TCM use is a Chinese government priority. *The Healthy China 2030 Program proposes* to develop TCM and conventional medicine equally, emphasizing the unique role of TCM in the prevention and treatment of major diseases, such as cancer. China’s Healthy *China action-implementation plan for cancer prevention and control (2019–2022)* highlights the action of integrating TCM and conventional medicine, accelerating the construction of a network of TCM cancer prevention, control, and treatment. In China, TCM has been widely used in lung cancer treatments. TCM could enhance conventional treatments (chemotherapy, radiation therapy, and epidermal growth factor receptors (EGFRs) tyrosine kinase inhibitors (TKIs)) effects as well as provide synergistic effects, enhance chemotherapy drugs chemosensitivity, reverse drug resistance, reduce adverse reactions and toxicity, relieve patients’ pain and improve quality of life [14]. After treating with TCM, lung cancer cells will induce apoptosis and/or autophagy, suppress metastasis, impact immune reaction, and therapeutic effect of EGFR-TKIs [14]. Li et al. identified 6939 lung cancer patients and collected 264 TCM users and 528 non-TCM users. The results demonstrated that three formulas (Xiang-Sha-Liu-Jun-Zi-Tang, Bu-Zhong-Yi-Qi-Tang, Bai-He-Gu-Jin-Tang) and three herbs (Bei-Mu, Ge-Gen, Xing-Ren) increased the efficacy and reduced the mortality hazard ratio in lung cancer [11]. Liao et al. performed a longitudinal study of over 100,000 newly diagnosed lung cancer patients. They found that TCM significantly reduced the risk of death by 32% compared to patients without TCM [15]. Both studies confirmed the efficacy of TCM in the treatment of lung cancer.

In addition to studies on the efficacy of TCM in the treatment of lung cancer, the issue of the cost of using TCM to treat lung cancer is also a key topic of research. However, few investigations have revealed whether increased use of TCM could reduce the utilization of conventional medicine or save families costly medical interventions. Using data on lung cancer patients from Taiwanese National Health Insurance database from 1997 to 2009, Liao et al. found that the expenditure, and the mortality, of TCM users was less than TCM nonusers in patients without surgery [16]. Using patient diagnosis and treatment data from 47 Beijing hospitals in 2019, Liu et al. found that the average times and daily costs of lung cancer inpatients were relatively low when using Chinese herbal medicine [17]. Meanwhile, there is a lack of nationwide research to evaluate TCM impact on the economic burden of lung cancer treatments in mainland China. To address this gap in the literature, our study evaluated whether the use of TCM reduces or increases the cost of treatment for lung cancer inpatients and further explored the reasons why TCM use affects the cost of treatment for lung cancer inpatients, and how to avoid unnecessary hospitalization costs when TCM is used. Our study provides information for health authorities to optimize TCM treatment for inpatients with lung cancer.

## Materials and methods

### Design and setting

This study is a cross-sectional study. Since the data sets we used were anonymized database and had no impact on patients’ health and care, the informed consent was exempted. This study was approved and the permission to access the raw data was granted by the Ethics Committee of Beijing University of Chinese Medicine (No.2019BZHYLL0201). All methods were carried out in accordance with relevant guidelines and regulations.

### Study population and sampling

The database used in this study was provided by the China Health Insurance Research Association (CHIRA). CHIRA performed the collection of the database, which covered more than 93% of China’s urban residents under the Urban Employee Basic Medical Insurance (UEBMI) and Urban Resident Basic Medical Insurance (URBMI) schemes.

The CHIRA claims database is only available for researchers up to 2017. Therefore, we requested access to claims data for lung cancer inpatients from 2010 to 2016 and randomly sampled 5% of the total data. The sampling principle was that each kth record was selected from the CHIRA population of size N, and the first sample record was selected from the random number table. In this way, a sample size of n was obtained, where N/n > =k. Anonymous patients were selected by their unique patient identification code, so all visit records for the selected patients were collected.

TCM methods in CHIRA claim database comprised Chinese herbal medicine, Chinese patent medicine, and Chinese medicine injection. In this study, inpatient TCM lung cancer users used any one or combination of the three TCM treatments while TCM nonusers used no TCM treatment.

### Data collection

We extracted information from the sample data that was relevant to this study, including demographic information (sex and age), types of medical insurance expenditure (URBMI and UEBMI), diagnosis (classified according to the International Classification of Diseases, 10th edition (ICD-10)), length of hospital stay (LOS), hospital-level (primary, secondary, and tertiary), economic region (east, west, and central), year and inpatient medical costs. Since disease severity of patients is an important factor affecting treatment costs, patients registered in the second and third diagnoses of lung cancer were manually marked, and the number of comorbidities was used as an indicator of disease severity. When data was missing or inconsistent, such as when the length of stay did not match the inpatient and discharge dates, the patient was deleted. Finally, 47,393 patients were included.

The total inpatient medical costs were the total costs obtained by summing conventional medicine medication costs, TCM costs, and nonpharmacy costs, comprising all other inpatients’ medical costs, such as surgery, diagnostic testing, and medical consumable costs. Every time a patient was hospitalized, an expense record was generated, with costs traced through a fixed and unique individual identification code. The cost index of each category in this study is the sum of the costs of each category in all inpatient records of the patient in the year calculated according to the patient identification code. The cost characteristic variables were truncated by 0.05 and 99.95% to exclude the influence of extreme values. The average exchange rate between USD and RMB in 2016 was adopted for currency value conversion: USD1 = RMB6.6423.

### Statistical analysis

All analyses were performed using STATA software version MP16.0. Two-sided *P* values less than 0.05 was considered statistically significant. The basic characteristics of the sample, such as age, sex, and insurance type, were analyzed by descriptive statistics and chi-square tests. Since the data on medical costs is distributed with positive skewness, the cost data were tested using the median with interquartile range (IQR) and Mann-Whitney test. After adjusting the confounding variables, such as length of hospital stay, age, sex, insurance type, number of hospitalizations, hospital level, region, number of comorbidities, and year of treatment, the logarithm of the total inpatient medical cost was set as the dependent variables, and whether the patient used TCM or not was set as the independent variable. The difference in the total inpatient medical cost between TCM users and TCM nonusers was quantified by ordinary least squares regression analysis. Further, the logarithm of the cost of TCM is set as the dependent variable, and the logarithm of the cost of conventional medication costs and the logarithm of nonpharmacy costs were set as independent variables, to investigate the relationship between the cost of TCM and the conventional medication costs as well as nonpharmacy costs with an ordinary least squares regression model.

## Results

### Basic characteristics

As shown in Table 1, 47,393 inpatients with lung cancer, of which 38,697 or 81.7% were TCM users and 8696 were TCM nonusers. Table 1 shows that 66.2% (31,371) were male, and the proportion of male TCM users (66.8%) was significantly higher than that of TCM nonusers (63.5%) (*P* < 0.001). The median age of the sample was 64 and 76.3% (36,149) were URBMI insured; 69.3% (32,845) were hospitalized in the tertiary hospital, with the proportion of TCM users (27.5%) in secondary hospitals significantly higher than that of TCM nonusers (21.5%) (*P* < 0.001). And 47.5% (18,389) of TCM users came from the eastern region and 27.2% from the western region. Most inpatients (74.8% or 35,432) had no second or third diagnosis information and among the TCM users, 12.2 (4705) inpatients had one, 9.9% (3844) had two and 3.0% (1153) had three or more other comorbidities. The average length of stay per visit of TCM users (13.9 days) was longer than that of TCM nonusers (9.8 days) (*P* < 0.001). In terms of years of admission, the proportion of TCM users was the highest in 2016 (20.8%) and the lowest in 2010 (8.0%).

**Table 1:** Sample characteristics of TCM users and TCM nonusers among lung cancer patients

Characteristics	Overall	TCM nonusers	TCM users	*P*-value
Sex, *n* (%)				
Male	31,371 (66.2)	5525 (63.5)	25,864 (66.8)	< 0.001
Female	16,022 (33.8)	3171 (36.5)	12,851 (33.2)	
Age (years), median (IQR)	64 (57–73)	63 (55–71)	65 (57–73)	< 0.001
Age group, *n* (%)				
0–39	1024 (2.2)	297 (3.4)	727 (1.9)	< 0.001
40–59	16,160 (34.1)	3297 (37.9)	12,863 (33.2)	
60–69	15,514 (32.7)	2714 (31.2)	12,800 (33.1)	
70–79	11,703 (24.7)	1907 (21.9)	9796 (25.3)	
≧80	2992 (6.3)	481 (5.5)	2511 (6.5)	
Insurance type, *n* (%)				
UEBMI	36,149 (76.3)	6665 (76.6)	29,484 (76.2)	0.370
URBMI	11,244 (23.7)	2031 (23.4)	9213 (23.8)	
Hospital level, *n* (%)				
Primary	2044 (4.3)	337 (3.9)	1707 (4.4)	< 0.001
Secondary	12,504 (26.4)	1869 (21.5)	10,635 (27.5)	
Tertiary	32,845 (69.3)	6490 (74.6)	26,355 (68.1)	
Region, *n* (%)				
East	23,334 (49.2)	4945 (56.9)	18,389 (47.5)	< 0.001
Central	11,655 (24.6)	1883 (21.7)	9772 (25.3)	
West	12,404 (26.2)	1868 (21.5)	10,536 (27.2)	
No of comorbidities, *n* (%)				
0	35,432 (74.8)	6437 (74.0)	28,995 (74.9)	0.001
1	5824 (12.3)	1119 (12.9)	4705 (12.2)	
2	4778 (10.1)	934 (10.7)	3844 (9.9)	
≧3	1359 (2.9)	206 (2.4)	1153 (3.0)	
ALOS per time	13.0	9.8	13.9	< 0.001
Year, *n* (%)				
2010	4009 (8.5)	911 (10.5)	3098 (8.0)	< 0.001
2011	5789 (12.2)	1398 (16.1)	4391 (11.4)	
2012	5307 (11.2)	1237 (14.2)	4070 (10.5)	
2013	7486 (15.8)	1371 (15.8)	6115 (15.8)	
2014	7254 (15.3)	1080 (12.4)	6174 (16.0)	
2015	7987 (16.9)	1181 (13.6)	6806 (17.6)	
2016	9561 (20.2)	1518 (17.5)	8043 (20.8)	
Number of patients, *n* (%)	47,393 (100.0)	8696 (18.3)	38,697 (81.7)	

*P* values are based on the chi-square test and Mann–Whitney test; *TCM* Traditional Chinese medicine, *UEBMI* Urban Employee Basic Medical Insurance scheme, *URBMI* Urban Resident Basic Medical Insurance scheme, *IQR* Interquartile range

### Inpatient medical cost between TCM users and TCM nonusers

Table 2 shows the difference in the total medical cost of lung cancer inpatients between TCM users and TCM nonusers. The median inpatient medical cost of TCM users was RMB18,798 (USD2,830), which was significantly higher than RMB8,001 (USD1,205) for TCM nonusers (*P* < 0.001). In terms of sex, age, insurance type, hospital level, region, and the number of comorbidities, the total inpatient medical cost of TCM users was significantly higher than that of TCM nonusers (all *P* < 0.001). From 2010 to 2016, the total inpatient medical cost of TCM users was also significantly higher than those of TCM nonusers (all *P* < 0.001).

**Table 2:** Inpatient medical cost of TCM users and TCM nonusers

Characteristics	TCM nonusers		TCM users		*P*-value
	Median	IQR	Median	IQR	
Sex					
Male	7954	(3854–17,612)	18,846	(9001–46,410)	< 0.001
Female	8084	(3741–19,210)	18,717	(8826–44,992)	< 0.001
Age group					
0–39	9614	(4154–28,107)	21,773	(9519–53,594)	< 0.001
40–59	8238	(3856–19,017)	19,853	(9245–51,669)	< 0.001
60–69	7954	(3763–18,483)	20,307	(9297–49,885)	< 0.001
70–79	7676	(3798–16,735)	17,307	(8521–38,743)	< 0.001
≧80	7606	(3683–13,720)	14,666	(7868–31,591)	< 0.001
Insurance type					
UEBMI	8606	(4025–19,596)	20,195	(9710–48,880)	< 0.001
URBMI	6599	(3291–13,818)	14,953	(6970–36,399)	< 0.001
Hospital level					
Primary	4040	(1738–10,944)	8232	(3932–19,044)	< 0.001
Secondary	5344	(2818–10,832)	14,542	(6877–34,815)	< 0.001
Tertiary	9384	(4491–20,769)	21,914	(10623–51,997)	< 0.001
Region					
East	8535	(3981–19,134)	20,000	(9671–49,113)	< 0.001
Central	7394	(3660–18,283)	21,453	(8906–51,666)	< 0.001
West	7465	(3700–15,296)	15,602	(7962–34,929)	< 0.001
No of comorbidities					
0	7994	(4130–16,145)	21,205	(9892–52,812)	< 0.001
1	12,860	(5582–32,844)	34,143	(14011–65,841)	< 0.001
2	14,504	(6252–27,992)	33,536	(15763–80,128)	< 0.001
≧3	15,695	(10412–32,255)	28,542	(12758–58,157)	< 0.001
Year					
2010	6734	(3739–12,144)	11,504	(6692–20,103)	< 0.001
2011	6571	(3250–12,738)	11,070	(6639–18,060)	< 0.001
2012	6755	(3603–14,353)	13,220	(7458–22,783)	< 0.001
2013	8615	(3440–20,621)	29,302	(12275–63,168)	< 0.001
2014	10,134	(4794–24,256)	25,559	(11272–55,516)	< 0.001
2015	8887	(4093–23,622)	24,136	(9527–59,265)	< 0.001
2016	10,060	(4280–28,678)	25,973	(10769–59,945)	< 0.001

*P* values are based on the Mann–Whitney test; TCM: traditional Chinese medicine; UEBMI: Urban Employee Basic Medical Insurance scheme; URBMI: Urban Resident Basic Medical Insurance scheme; IQR: Interquartile range

### Analysis of total inpatient medical costs of TCM users and TCM nonusers

To further analyze the difference in total inpatient medical cost between TCM users and TCM nonusers, we conducted a multivariate regression analysis. As shown in Table 3, after the confounding variables were fixed, the use of TCM was 65.2% higher than that for TCM nonusers (Coef. = 0.502, *P* < 0.001), with TCM users incurring a significantly heavier medical burden than TCM nonusers.

**Table 3:** Multiple regression analysis for total inpatient costs

Parameters	Coef.	*P* > *z*	95% Wald confidence interval	
			Lower	Upper
Use of TCM				
TCM user	0.502	< 0.001	0.483	0.521
Length of stay	0.023	< 0.001	0.023	0.023
Age	−0.004	< 0.001	− 0.004	− 0.003
Sex				
Male	0.006	0.412	−0.009	0.022
Insurance type				
UEBMI	0.190	< 0.001	0.173	0.208
No of hospitalizations	0.046	< 0.001	0.040	0.051
Hospital type				
Secondary hospital	0.233	< 0.001	0.196	0.271
Tertiary hospital	0.640	< 0.001	0.604	0.676
Region				
Central	−0.234	< 0.001	− 0.253	− 0.215
West	−0.172	< 0.001	− 0.190	− 0.153
No of comorbidities	0.061	< 0.001	0.055	0.068
Year				
2011	0.020	0.222	−0.012	0.052
2012	0.137	< 0.001	0.104	0.170
2013	0.276	< 0.001	0.245	0.307
2014	0.425	< 0.001	0.393	0.457
2015	0.419	< 0.001	0.388	0.451
2016	0.472	< 0.001	0.442	0.503
_Cons	7.993	< 0.001	7.930	8.056

R-square = 0.5419 and adjusted R-square = 0.5418 in a multiple linear regression model that was adjusted for the length of stay, age, sex, insurance type, number of hospitalizations, hospital level, region, number of comorbidities, and year of treatment. The baseline represents the inpatient cost for a female who did not use any TCM with Urban Resident Basic Medical Insurance admitted to a primary hospital in the eastern region without any comorbidity. *TCM* Traditional Chinese medicine, *UEBMI* Urban Employee Basic Medical Insurance scheme; *URBM*I Urban Resident Basic Medical Insurance scheme

### Composition of total inpatient costs for TCM users and TCM nonusers

To identify where TCM users faced a significantly higher medial cost burdened than TCM nonusers, we conducted a composition analysis of total inpatient costs. Based on the classification of the insurance payment system, total costs were divided into conventional medication costs, TCM costs, and nonpharmacy costs. Table 4 shows that medication cost, conventional medication cost, and nonpharmacy cost of TCM users were all higher than TCM nonusers, revealing the higher medical cost of TCM users is not done only to TCM use.

**Table 4:** Composition of medication and medical cost of TCM users and TCM nonusers

Variables	TCM nonusers	TCM users	*P*-value
Total medical cost			
Median	8001	18,798	< 0.001
IQR	3826–18,140	8947–45,927	
Total medication cost			
Median	3032	10,274	< 0.001
IQR	858–7327	4691–22,019	
Percentage	40.0	79.5	
Conventional medication cost			
Median	3032	7384	< 0.001
IQR	858–7327	3057–16,789	
Percentage	40.0	69.3	
TCM cost			
Median	–	1951	< 0.001
IQR	–	626–4942	
Percentage	–	10.2	
Nonpharmacy cost			
Median	3633	7302	< 0.001
IQR	1611–9441	3070–20,572	
Percentage	60.0	20.5	

*P* values are based on the Mann–Whitney test; *TCM* Traditional Chinese medicine, *IQR* Interquartile range

### Multiple regression analysis to test the correlation between TCM cost

The correlation between TCM costs and conventional medication costs as well as nonpharmacy costs could contribute to further explaining why TCM users endure greater economic medical burden. Table 5 shows that there was a positive correlation between TCM costs, conventional medication costs (Coef. = 0.283, *P* < 0.001), and nonpharmacy costs (Coef. = 0.211, *P* < 0.001). With confounding factors fixed, TCM cost had a positive correlation with conventional medication cost as well as nonpharmacy cost, indicting synchronous increase of TCM costs and conventional medication cost for TCM users. The use of TCM did not offset the costs of using conventional medicine. TCM treatment played a role of complement, but not substitute, for conventional medicine.

**Table 5:** Multiple regression analysis for TCM cost

Parameters	Coef.	*P* > *z*	95% Wald confidence interval	
			Lower	Upper
***Model 1***				
Conventional medication cost	0.283	< 0.001	0.268	0.298
Length of stay	0.013	< 0.001	0.012	0.014
Age	0.003	< 0.001	0.001	0.004
Sex				
Male	0.009	0.589	−0.023	0.041
Insurance type				
UEBMI	−0.298	< 0.001	−0.336	− 0.261
No of hospitalizations	0.085	< 0.001	0.074	0.095
Hospital type				
Secondary hospital	0.335	< 0.001	0.257	0.413
Tertiary hospital	0.545	< 0.001	0.470	0.620
Region				
Central	0.191	< 0.001	0.151	0.231
West	0.182	< 0.001	0.143	0.220
No of comorbidities	−0.014	0.037	−0.027	−0.001
Year				
2011	0.093	0.009	0.023	0.163
2012	0.180	< 0.001	0.109	0.251
2013	0.028	0.417	−0.039	0.095
2014	0.223	< 0.001	0.155	0.291
2015	0.263	< 0.001	0.196	0.330
2016	0.191	< 0.001	0.125	0.256
_Cons	3.424	< 0.001	3.248	3.600
***Model 2***				
Nonpharmacy cost	0.211	< 0.001	0.194	0.227
Length of stay	0.014	< 0.001	0.013	0.015
Age	0.001	0.073	0.000	0.003
Sex				
Male	−0.013	0.441	−0.046	0.020
Insurance type				
UEBMI	−0.314	< 0.001	− 0.352	− 0.277
No of hospitalizations	0.119	< 0.001	0.108	0.129
Hospital type				
Secondary hospital	0.290	< 0.001	0.210	0.369
Tertiary hospital	0.548	< 0.001	0.471	0.624
Region				
Central	0.150	< 0.001	0.109	0.190
West	0.175	< 0.001	0.137	0.214
No of comorbidities	−0.014	0.036	−0.028	−0.001
Year				
2011	0.095	0.008	0.025	0.166
2012	0.166	< 0.001	0.094	0.238
2013	−0.052	0.136	−0.121	0.016
2014	0.100	0.005	0.030	0.170
2015	0.146	< 0.001	0.077	0.215
2016	0.048	0.161	−0.019	0.116
_Cons	4.166	< 0.001	3.988	4.344

^a^R-square = 0.2559 and adjusted R-square = 0.2556 / R-square = 0.2403 and adjusted R-square = 0.2400. The baseline represents the inpatient cost for a female who did not use any TCM with Urban Resident Basic Medical Insurance admitted to a primary hospital in the eastern region without any comorbidity. *TCM* Traditional Chinese medicine, *UEBMI* Urban Employee Basic Medical Insurance scheme, *URBMI* Urban Resident Basic Medical Insurance scheme

## Discussion

Since most lung cancer patients are likely to succumb within 5 years of diagnosis [18], patients, their families and carers seek a wide range of treatments, including TCM [19, 20]. Our data show that between 2010 to 2016 the proportion of TCM users gradually increased, outweighing TCM nonusers, perhaps related to the Chinese government’s emphasis on TCM, including preferential policies for TCM hospitals.

Whether TCM can reduce the cost of disease treatment has been the subject of much research interest. Using a Taiwanese National Health Insurance database, Su et al. found that, among patients with uterine fibroid, TCM use correlated negatively with consumption of conventional medicine and decreased total medical costs [21]. Tsai et al. also demonstrated that, among patients with heart failure, the hospital cost was lower for TCM users than for TCM nonusers [22]. These two studies suggest that TCM can reduce healthcare costs in the treatment of uterine fibroids and heart failure. However, our study using medical insurance data for lung cancer in mainland China did not find similar findings. We found that the total inpatient cost of TCM users was higher than those of TCM nonusers controlling for demographic characteristics, a finding consistent with the study by Huang et al. for ischemic stroke [23]. This seems to be contrary to previous research that demonstrated CAM was generally cheaper than conventional medication, and that the use of CAM could reduce the economic burden of disease [24, 25]. We found that the median cost of conventional medicine medication costs for TCM users was RMB7,384 (USD1,112), which is significantly higher than RMB3,032 (USD456) for TCM nonusers. Similarly, the median nonpharmacy cost of TCM users was RMB7,302 (USD1,099), which was also significantly higher than that of TCM nonusers (RMB3,363 / USD506). The medication, conventional medicine medication, and nonpharmacy costs of TCM users was all higher than TCM nonusers, which shows that higher medical cost of TCM users was not just done to TCM treatments. One possible explanation is that the disease status of TCM users was more serious than TCM nonusers, so all kinds of medical services were consumed more by TCM users than TCM nonusers [26]. With confounding factors fixed, regression results show that TCM costs had a positive correlation with conventional medication cost as well as nonpharmacy cost, indicting synchronous increase of TCM cost, conventional medication cost, and nonpharmacy cost for TCM users. Our data show that the use of TCM did not reduce the use of conventional medicine. Given the large gap between the total cost of traditional Chinese medicine (10.2%) and the cost of conventional drugs (69.3%), TCM treatment mainly plays a complementary role rather than a substitute role.

There are several possible reasons why the TCM, conventional medicine medication, and nonpharmacy cost for TCM users increase simultaneously. First, TCM users also used conventional medicine treatments, so TCM users faced more treatments than TCM nonusers. Second, TCM users might have a more serious disease status than TCM nonusers, then they have to utilize more medical services, including both TCM services and conventional medical services. Clinical studies show that surgical resection of lesions and other lung cancer treatment methods cause significant body damage and a variety of side effects, such as liver and kidney damage, decreasing immunity, multiple infections, vomiting, and diarrhea [6–8, 27, 28], so patients and doctors might use TCM to reduce the side effects of conventional medicine treatment [29, 30]. Third, TCM higher costs might also be related to health providers’ profit-seeking behavior. Medical service providers were allowed to add a mark-up on all non-TCM drug sales, usually, about 15%, which increased hospital and doctor income and encouraged over-prescribing, before the drug zero-markup policy came into effect in all hospital in 2017 [31]. Finally, some patients may seek excessive treatments [32, 33].

When both TCM and conventional medicine play an active role in clinical diagnosis and treatment, doctors need to consider when TCM treatments and conventional medicine treats complement or substitute for each other. In this case, using either conventional medicine or TCM treatments, but not both, would control treatment costs. Doctors need training on when TCM and conventional medicine can be substitutes, and patients also need to be informed about the choices between TCM and conventional medicine treatments. For the treatment of lung cancer, there are relatively clear clinical guidelines for the use of conventional drugs, but not TCM drug treatment [34, 35]. Given that the Chinese government re-emphasized in 2021 that it “attached equal importance to TCM and Western medicine”, we recommend a joint Clinical Guideline to codify the use of TCM and conventional medicine for lung cancer treatment. Such a codification would inform the treatment plan for patients, attaining an appropriate balance between TCM and conventional medicine treatments. According to patients’ actual needs, TCM and conventional medicine should reflect a reasonable substitution relationship, which can not only give full play to the therapeutic role of integrated TCM and conventional medicine but also control the economic medical burden on patients.

There are some limitations in the present study. First, the CHIRA database did not include efficacy indicators for clinical treatment and could not provide evidence on the actual efficacy and clinical outcomes. The database contained limited personal information about patients, which resulted in the inability to consider the influence of factors such as the economic level and education of patients. Second, TCM hospitals were the main body for TCM treatment service provision, but the CHIRA marker for hospital category did not include this information. Future research should collect information from TCM and non-TCM hospitals. The CHIRA database did not provide information on the stage of lung cancer, so we are unable to analyze the cost differences caused by different treatments for different lung cancer stages. Future research should collect data on the lung cancer stage. Third, acupuncture and massage are also important components of TCM treatment, but the cost information of both was recorded in the nonpharmacy costs and cannot be identified individually. Our study can only attribute acupuncture and massage to nonmedication therapy services. Finally, the coverage years of our data were from 2010 to 2016 due to the CHIRA limitations on post-2016 access. In China’s fragmented insurance system, UEBMI and URBMI were the two schemes for the urban population until an amalgamation of URBMI and New Rural Cooperative Medical Scheme (NCMS) beginning in 2017 but currently incomplete. Importantly, the amalgamation of NCMS with URBMI aimed to upgrade rural benefits and coverage to the same level as URBMI. As a result, the CHIRA data are consistent with current URBMI, as well as UEBMI, benefits, and coverage.

## Conclusions

Inpatient TCM users incurred a heavier lung cancer treatment cost burden than inpatient TCM nonusers. In addition, TCM users’ conventional medicine medication cost and nonpharmacy cost were also higher than TCM nonusers, which indicates that the higher medical costs of TCM users were due to both TCM and conventional medicine treatments. Importantly, these cost data indicates that TCM mainly played a complementary, rather than an alternative to conventional medicine, role. We suggest that a joint Clinical Guideline that could balance the use of TCM and conventional medicine should be developed. With the norms and guidance of joint clinical guidelines, the advantages of traditional Chinese medicine and conventional medicine can be brought into full play, and patients can not only enjoy the best curative effect, but also bear less economic burden of lung cancer treatment.

## Data Availability

The data that support the findings of this study are available from China Health Insurance Research Association but restrictions apply to the availability of these data, which were used under license for the current study and so are not publicly available. Data are however available from the authors upon reasonable request and with permission of the China Health Insurance Research Association.

## Abbreviations

TCM: Traditional Chinese medicine
DALYs: Disability-adjusted life-years
CAM: Complementary and alternative medicine
CHIRA: China Health Insurance Research Association
UEBMI: Urban Employee Basic Medical Insurance
URBMI: Urban Resident Basic Medical Insurance
LOS: Length of stay
IQR: Interquartile range

## References

[1] Sung H, Ferlay J, Siegel RL, Laversanne M, Soerjomataram I, Jemal A (2021). Global Cancer statistics 2020: GLOBOCAN estimates of incidence and mortality worldwide for 36 cancers in 185 countries. CA Cancer J Clin.

[2] Qiu H, Cao S, Xu R (2021). Cancer incidence, mortality, and burden in China: a time-trend analysis and comparison with the United States and United Kingdom based on the global epidemiological data released in 2020. Cancer Commun (Lond).

[3] Cao W, Chen HD, Yu YW, Li N, Chen WQ (2021). Changing profiles of cancer burden worldwide and in China: a secondary analysis of the global cancer statistics 2020. Chin Med J.

[4] Park J, Look KA (2019). Health care expenditure burden of cancer care in the United States. Inquiry.

[5] Sun CY, Shi JF, Fu WQ, Zhang X, Liu GX, Chen WQ (2021). Catastrophic health expenditure and its determinants in households with lung cancer patients in China: a retrospective cohort study. BMC Cancer.

[6] Lemjabbar-Alaoui H, Hassan OU, Yang YW, Buchanan P (2015). Lung cancer: biology and treatment options. Biochim Biophys Acta.

[7] Vinod SK, Hau E (2020). Radiotherapy treatment for lung cancer: current status and future directions. Respirology.

[8] Cagle PT, Chirieac LR (2012). Advances in treatment of lung cancer with targeted therapy. Arch Pathol Lab Med.

[9] Molassiotis A, Fernandez-Ortega P, Pud D, Ozden G, Scott JA, Panteli V (2005). Use of complementary and alternative medicine in cancer patients: a European survey. Ann Oncol.

[10] Luo Q, Asher GN (2017). Complementary and alternative medicine use at a Comprehensive Cancer Center. Integr Cancer Ther.

[11] Li TM, Yu YH, Tsai FJ, Cheng CF, Wu YC, Ho TJ (2018). Characteristics of Chinese herbal medicine usage and its effect on survival of lung cancer patients in Taiwan. J Ethnopharmacol.

[12] Li SG, Chen HY, Ou-Yang CS, Wang XX, Yang ZJ, Tong Y (2013). The efficacy of Chinese herbal medicine as an adjunctive therapy for advanced non-small cell lung cancer: a systematic review and meta-analysis. PLoS One.

[13] Sun H, Zhang L, Sui B, Li Y, Yan J, Wang P (2021). The effect of Terpenoid natural Chinese medicine molecular compound on lung Cancer treatment. Evid Based Complement Alternat Med.

[14] Su XL, Wang JW, Che H, Wang CF, Jiang H, Lei X (2020). Clinical application and mechanism of traditional Chinese medicine in treatment of lung cancer. Chin Med J.

[15] Liao YH, Li CI, Lin CC, Lin JG, Chiang JH, Li TC (2017). Traditional Chinese medicine as adjunctive therapy improves the long-term survival of lung cancer patients. J Cancer Res Clin Oncol.

[16] Liao YH, Lin JG, Lin CC, Li TC (2013). Distributions of usage and the costs of conventional medicine and traditional chinese medicine for lung cancer patients in Taiwan. Evid Based Complement Alternat Med.

[17] Liu L, Yu J, Wang Q, Yang Y, Li S, Xu Y (2021). Research on the participation rate and costs of traditional Chinese medicine for lung Cancer based on real world study. Chinese Health Econ.

[18] Jones GS, Baldwin DR (2018). Recent advances in the management of lung cancer. Clin Med (London, England).

[19] Oyunchimeg B, Hwang JH, Ahmed M, Choi S, Han D (2017). Complementary and alternative medicine use among patients with cancer in Mongolia: a national hospital survey. BMC Complement Altern Med.

[20] Bahall M (2017). Prevalence, patterns, and perceived value of complementary and alternative medicine among cancer patients: a cross-sectional, descriptive study. BMC Complement Altern Med.

[21] Su SY, Muo CH, Morisky DE (2015). Use of Chinese medicine correlates negatively with the consumption of conventional medicine and medical cost in patients with uterine fibroids: a population-based retrospective cohort study in Taiwan. BMC Complement Altern Med.

[22] Tsai MY, Hu WL, Chiang JH, Huang YC, Chen SY, Hung YC (2017). Improved medical expenditure and survival with integration of traditional Chinese medicine treatment in patients with heart failure: a nationwide population-based cohort study. Oncotarget.

[23] Huang Z, Shi X, Nicholas S, Maitland E, Yang Y, Zhao W (2021). Use of traditional Chinese medicine and its impact on medical cost among urban ischemic stroke inpatients in China: a National Cross-Sectional Study. Evid Based Complement Alternat Med.

[24] Lu WI, Lu DP (2014). Impact of chinese herbal medicine on american society and health care system: perspective and concern. Evid Based Complement Alternat Med.

[25] Herman PM, Craig BM, Caspi O (2005). Is complementary and alternative medicine (CAM) cost-effective? A systematic review. BMC Complement Altern Med.

[26] Ding R, Zhu D, He P, Ma Y, Chen Z, Shi X (2020). Comorbidity in lung cancer patients and its association with medical service cost and treatment choice in China. BMC Cancer.

[27] Islam KM, Anggondowati T, Deviany PE, Ryan JE, Fetrick A, Bagenda D (2019). Patient preferences of chemotherapy treatment options and tolerance of chemotherapy side effects in advanced stage lung cancer. BMC Cancer.

[28] Williams PA, Cao S, Yang D, Jennelle RL (2020). Patient-reported outcomes of the relative severity of side effects from cancer radiotherapy. Support Care Cancer.

[29] Yao J, Jiao L, Yao Y, Lu Y, Shi J, Li J (2020). The effect of comprehensive rehabilitation program plus chemotherapy on quality of life in patients with postoperative non-small-cell lung cancer: study protocol of a multicenter randomized clinical trial. Trials.

[30] Lu CL, Li X, Zhou HM, Zhang C, Yang YY, Feng RL (2021). Traditional Chinese medicine in Cancer care: an overview of 5834 randomized controlled trials published in Chinese. Integr Cancer Ther.

[31] Zhu D, Shi X, Nicholas S, Bai Q, He P (2019). Impact of China's healthcare price reforms on traditional Chinese medicine public hospitals in Beijing: an interrupted time-series study. BMJ Open.

[32] Jin L (2010). From mainstream to marginal? Trends in the use of Chinese medicine in China from 1991 to 2004. Soc Sci Med.

[33] Zhu D, Shi X, Nicholas S, Ma Y, He P (2021). Estimated annual prevalence, medical service utilization and direct costs of lung cancer in urban China. Cancer Med.

[34] Ettinger DS, Wood DE, Aisner DL, Akerley W, Bauman JR, Bharat A (2021). NCCN guidelines insights: non-small cell lung cancer, version 2.2021. J Natl Compr Cancer Netw.

[35] Domine M, Moran T, Isla D, Marti JL, Sullivan I, Provencio M (2020). SEOM clinical guidelines for the treatment of small-cell lung cancer (SCLC) (2019). Clin Transl Oncol.

